# Supplementary material for: The Association of the COVID-19 Pandemic with the Uptake of Colorectal Cancer Screening Varies by Socioeconomic Status in Flanders, Belgium
Source: Cancers (Basel). 2024 Nov 27;16(23):3983. doi: 10.3390/cancers16233983 (PMC11640482; doi:10.3390/cancers16233983)
Supplement: Supplementary file 1 [file cancers-16-03983-s001.zip › cancers-3304173-supplementary.pdf]

Supplementary Materials

Supplementary Figures and Tables for Statistical Analysis

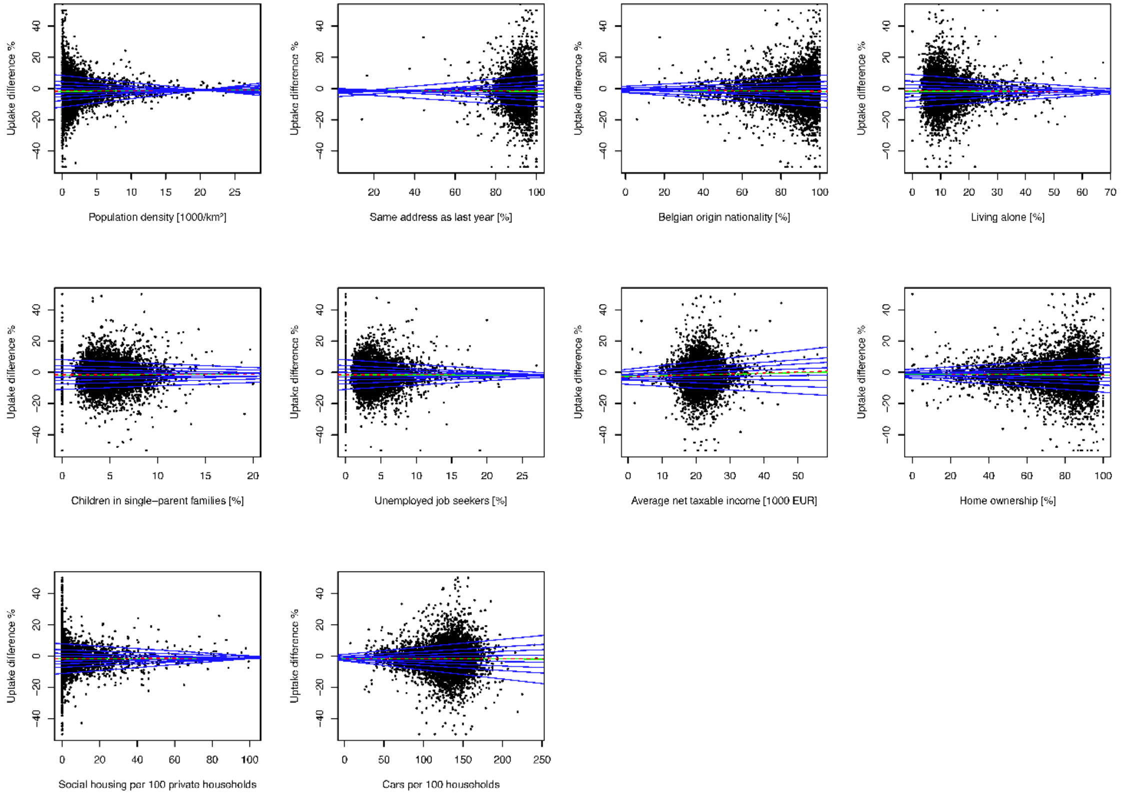

Figure S1. Univariate regression 10-quantile fit curves of all variables on difference in uptake rate of colorectal cancer screening program between the period during COVID-19 and the period before COVID-19 among statistical sectors in Flanders, Belgium. Footnotes: Blue lines are the 10-quantile fit curves; green lines are the 0.5 quantile regression fit curves; red lines are the linear regression fit curves.

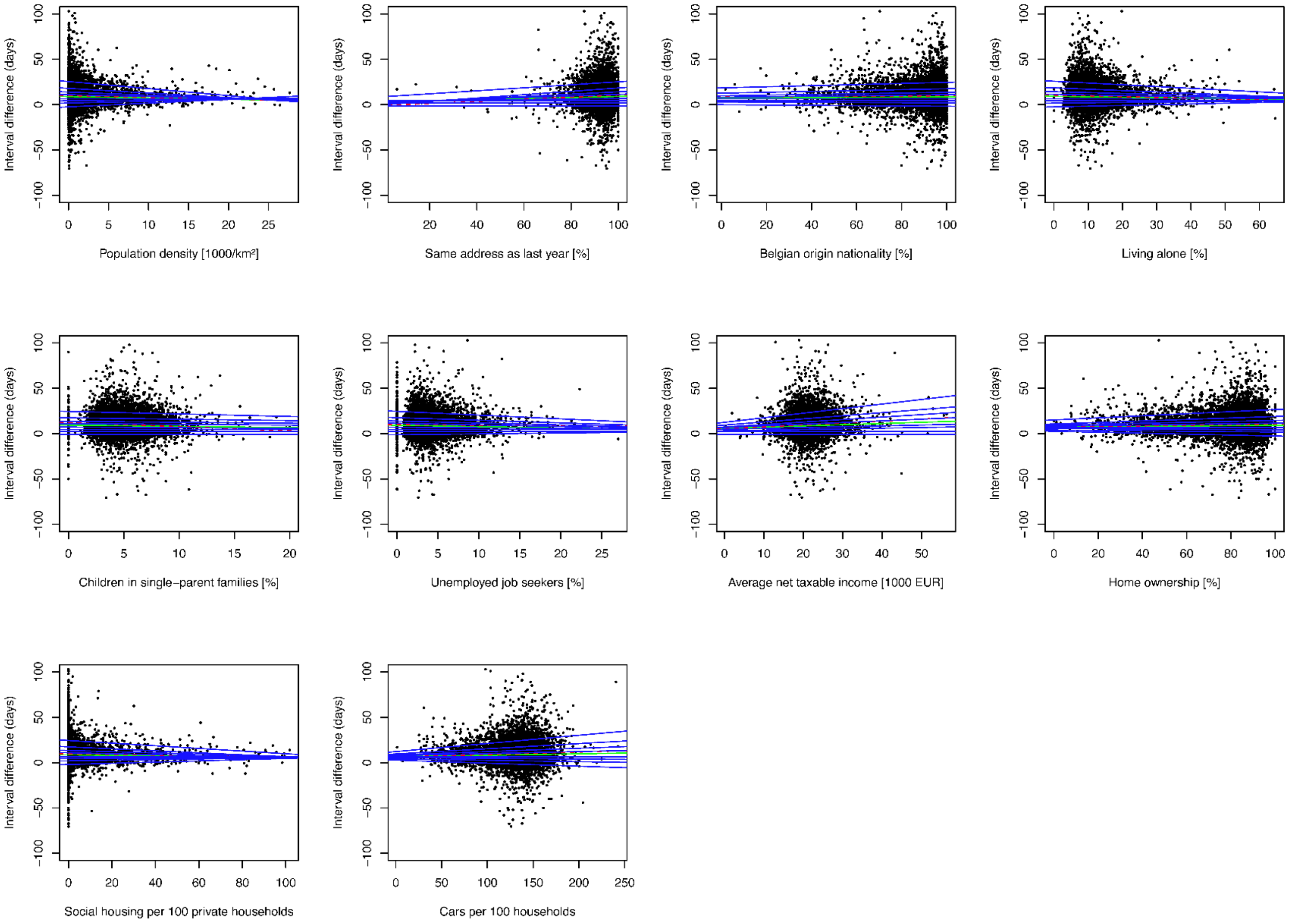

Figure S2. Univariate regression 10-quantile fit curves of all variables on difference in screening interval of colorectal cancer screening program between the period during COVID-19 and the period before COVID-19 among statistical sectors in Flanders, Belgium. Footnotes: Blue lines are the 10-quantile fit curves; green lines are the 0.5 quantile regression fit curves; red lines are the linear regression fit curves.

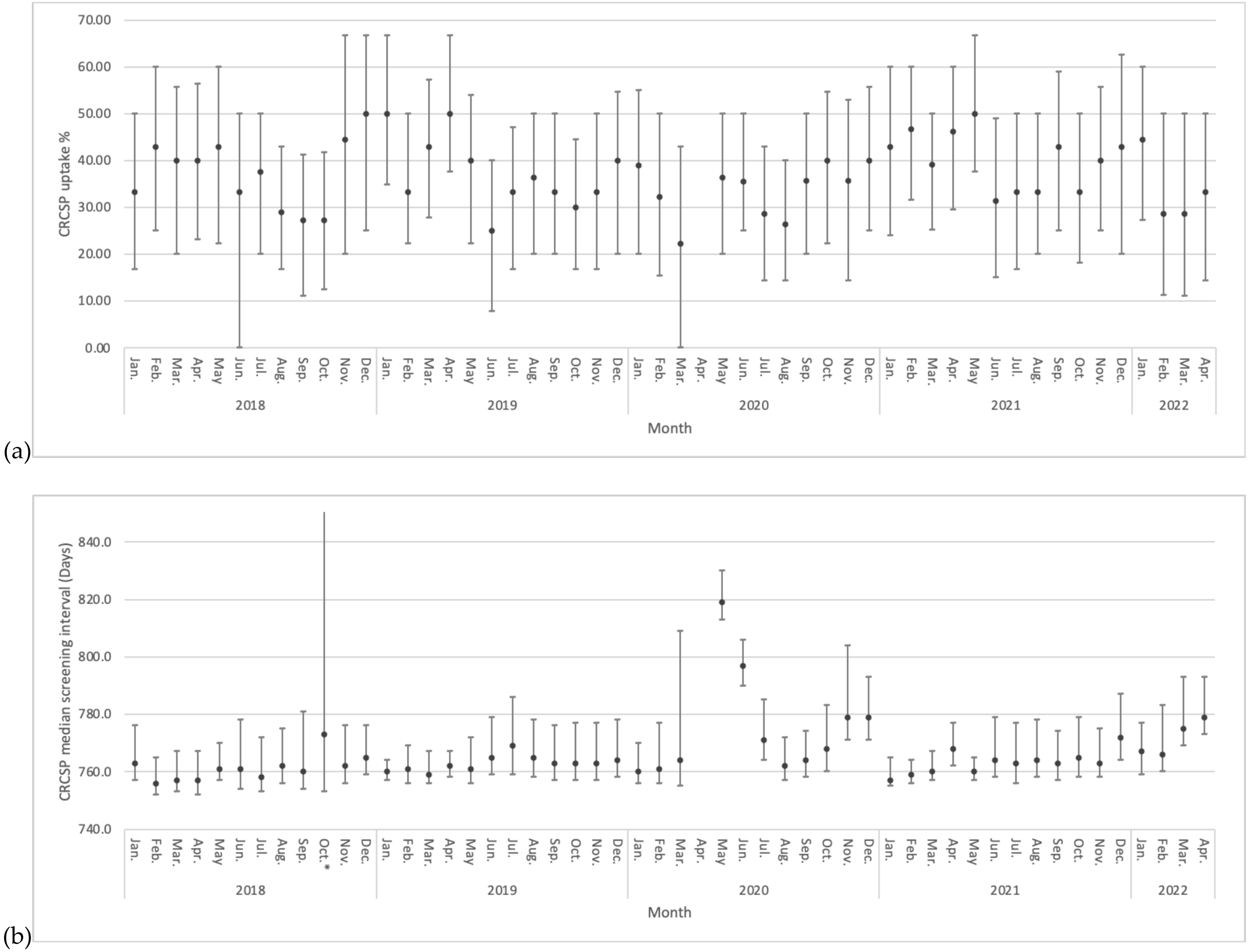

Figure S3. (a) Short-term uptake rate of the colorectal cancer screening program per month; (b) median screening interval of the colorectal cancer screening program per month among statistical sectors in Flanders, Belgium, during 2018-2022. Footnotes: The black dots are the median value, and the dark grey bars are the inter-quartile ranges. * The 75th quantile of Median screening interval in October 2018 was 912.3 days. CRCSP: Colorectal cancer screening program.

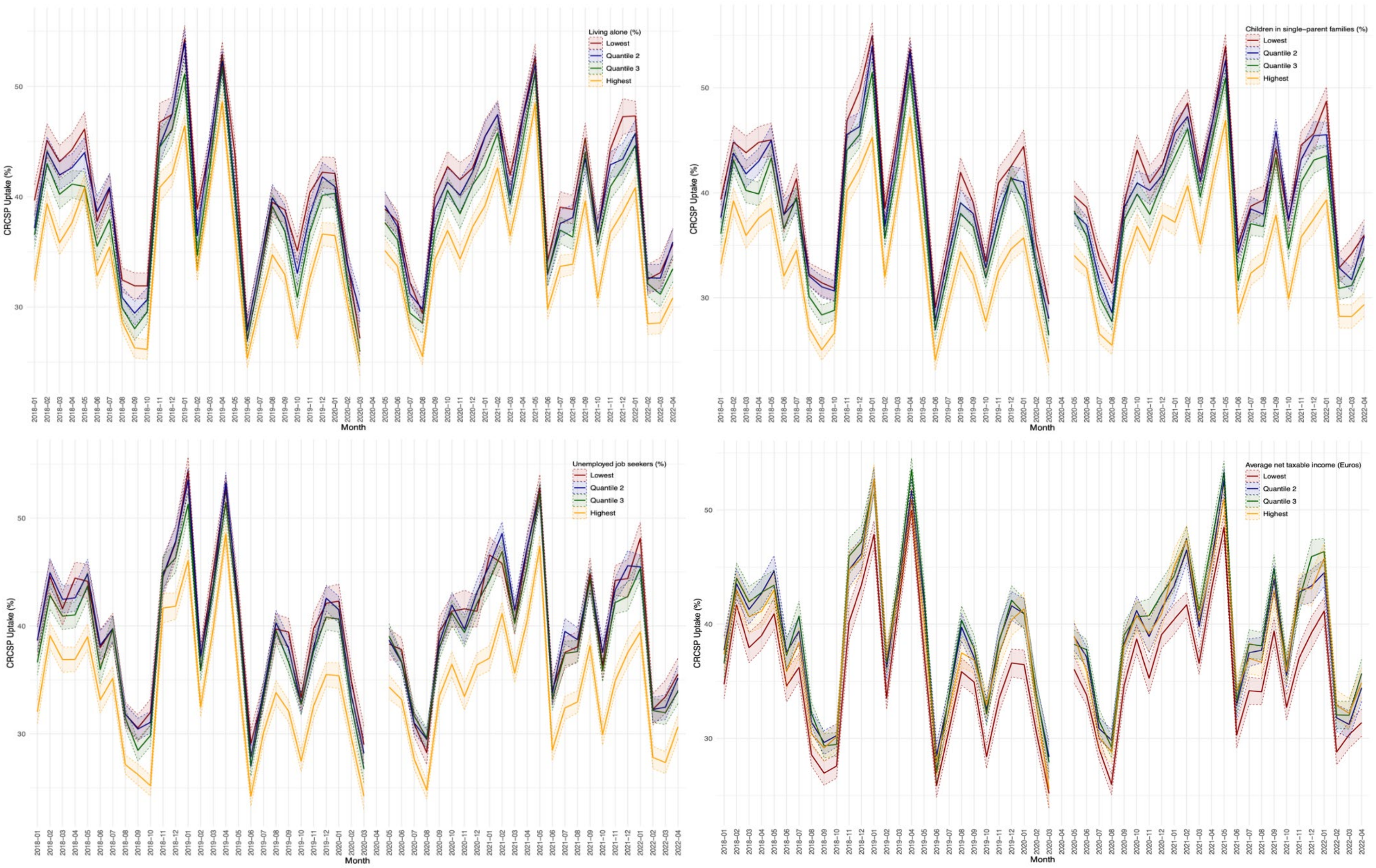

Figure S4. Monthly trends in screening uptake rate of the colorectal cancer screening program by quartile of the socioeconomic status variables (Living alone, Children in single-parent families, Unemployed job seekers, and Average net taxable income) among statistical sectors in Flanders, Belgium, during 2018-2022.

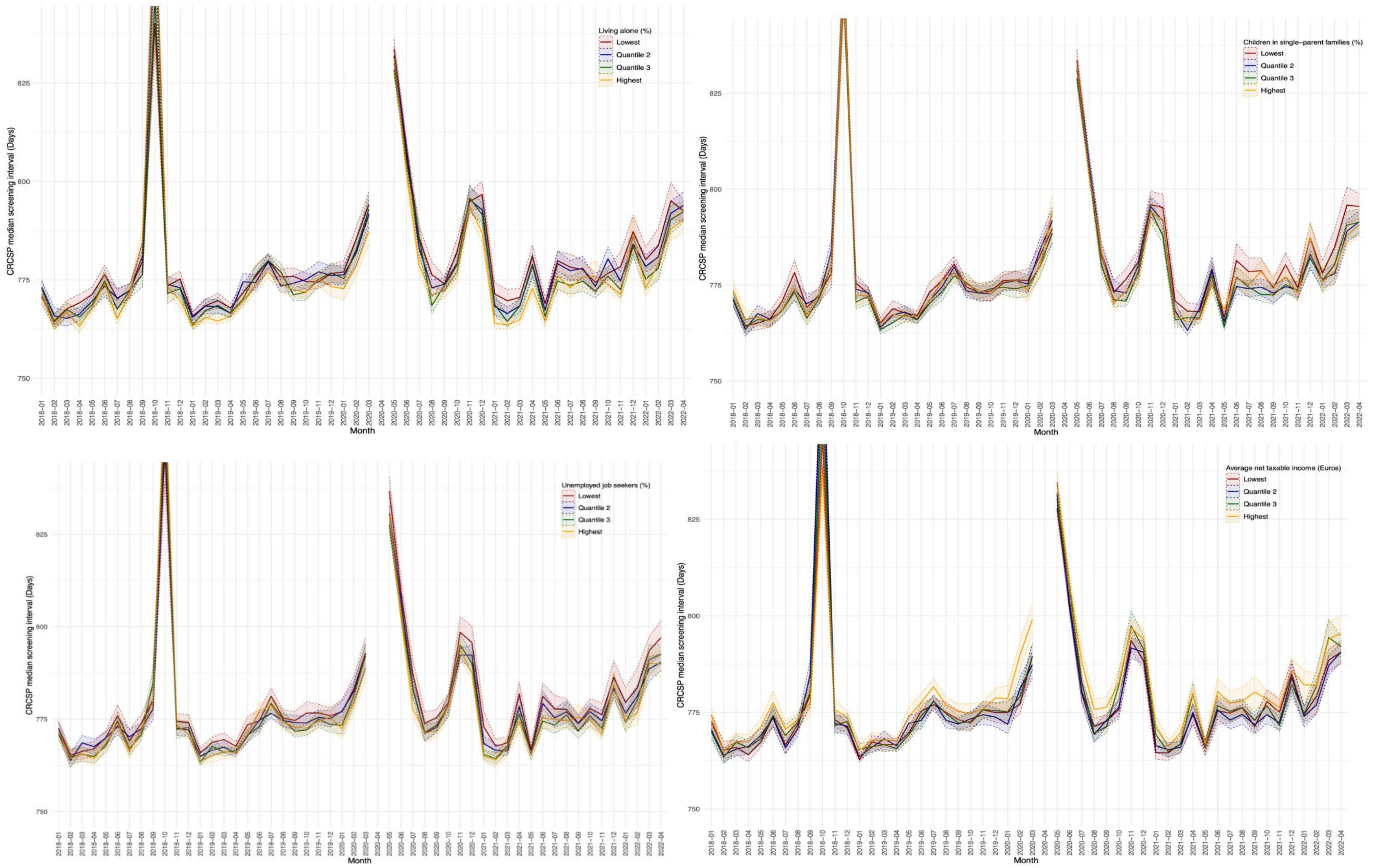

Figure S5. Monthly trends in screening interval of the colorectal cancer screening program by quartile of the socioeconomic status variables (Living alone, Children in single-parent families, Unemployed job seekers, and Average net taxable income) among statistical sectors in Flanders, Belgium, during 2018-2022.

Table S1. Coefficients of multivariable quantile regression of all variables on different quantiles of difference in uptake rate of colorectal cancer screening program between the period during COVID-19 and the period before COVID-19 among statistical sectors in Flanders, Belgium [$].

Variables	Q10	Q20	Q30	Q40	Q50	Q60	Q70	Q80	Q90
Population density, 1000/km²	0.43	0.33	0.23	0.16	0.09	0.02	-0.05	-0.13	-0.29
95% CI	(0.29, 0.57)	(0.25, 0.41)	(0.18, 0.29)	(0.09, 0.23)	(0.03, 0.14)	(-0.05, 0.09)	(-0.11, 0.02)	(-0.21, -0.05)	(-0.42, -0.16)
p value	<0.001	<0.001	<0.001	<0.001	<0.01	0.60	0.18	<0.01	<0.001
Same address as last year, %	0.12	0.03	-0.01	0.01	0.02	0.00	-0.05	-0.03	0.05
95% CI	(0.00, 0.24)	(-0.06, 0.11)	(-0.08, 0.06)	(-0.03, 0.05)	(-0.03, 0.06)	(-0.07, 0.07)	(-0.09, -0.01)	(-0.08, 0.03)	(-0.06, 0.15)
p value	0.06	0.56	0.75	0.61	0.47	0.97	0.01	0.39	0.39
Belgian origin nationality, %	-0.01	0.00	-0.01	-0.01	-0.02	-0.01	0.00	0.00	0.01
95% CI	(-0.04, 0.03)	(-0.03, 0.03)	(-0.03, 0.02)	(-0.03, 0.00)	(-0.03, 0.00)	(-0.03, 0.01)	(-0.02, 0.02)	(-0.02, 0.02)	(-0.03, 0.05)
p value	0.73	0.99	0.54	0.16	0.06	0.60	0.79	0.98	0.55
Living alone, %	-0.09	-0.09	-0.06	-0.05	-0.04	-0.06	-0.06	-0.06	-0.09
95% CI	(-0.20, 0.01)	(-0.16, -0.02)	(-0.11, -0.01)	(-0.10, 0.00)	(-0.07, -0.01)	(-0.11, -0.01)	(-0.11, 0.00)	(-0.11, -0.01)	(-0.20, 0.02)
p value	0.08	<0.01	0.02	0.07	0.02	0.01	0.04	0.02	0.12
Children in single-parent families, %	0.10	-0.08	-0.03	0.00	0.06	0.01	-0.01	0.01	0.01
95% CI	(-0.08, 0.28)	(-0.20, 0.04)	(-0.15, 0.09)	(-0.10, 0.10)	(-0.03, 0.16)	(-0.09, 0.11)	(-0.13, 0.10)	(-0.12, 0.14)	(-0.18, 0.20)
p value	0.28	0.18	0.63	0.96	0.17	0.82	0.80	0.89	0.90
Unemployed job seekers, %	-0.10	-0.11	-0.08	-0.06	-0.08	-0.01	0.02	0.07	0.09
95% CI	(-0.29, 0.09)	(-0.24, 0.01)	(-0.18, 0.01)	(-0.16, 0.05)	(-0.16, 0.00)	(-0.12, 0.10)	(-0.10, 0.13)	(-0.05, 0.20)	(-0.14, 0.32)
p value	0.31	0.08	0.08	0.27	0.05	0.85	0.75	0.22	0.45
Average net taxable income, 1000€	0.09	0.05	0.07	0.07	0.10	0.08	0.10	0.10	0.12
95% CI	(-0.02, 0.20)	(-0.03, 0.13)	(0.01, 0.14)	(0.02, 0.13)	(0.04, 0.15)	(0.02, 0.15)	(0.05, 0.16)	(0.03, 0.17)	(0.00, 0.24)
p value	0.10	0.26	0.03	0.01	<0.001	0.02	<0.001	<0.01	0.05
Home ownership, %	-0.05	-0.06	-0.04	-0.04	-0.03	-0.03	0.00	0.00	-0.03
95% CI	(-0.10, 0.00)	(-0.10, -0.03)	(-0.07, -0.02)	(-0.06, -0.02)	(-0.05, 0.00)	(-0.05, 0.00)	(-0.03, 0.02)	(-0.03, 0.03)	(-0.08, 0.02)
p value	0.07	<0.001	<0.01	<0.001	0.02	0.04	0.81	0.93	0.20
Social housing per 100 private households	0.00	0.00	0.00	0.00	0.00	-0.01	0.01	-0.01	-0.03
95% CI	(-0.04, 0.04)	(-0.02, 0.03)	(-0.02, 0.03)	(-0.02, 0.02)	(-0.01, 0.02)	(-0.02, 0.01)	(-0.01, 0.02)	(-0.03, 0.02)	(-0.06, 0.00)
p value	0.89	0.97	0.70	0.74	0.75	0.64	0.54	0.50	0.09
Cars per 100 households	-0.01	0.01	0.01	0.02	0.01	0.01	0.00	0.00	-0.01
95% CI	(-0.04, 0.03)	(-0.01, 0.03)	(-0.01, 0.03)	(0.00, 0.03)	(-0.01, 0.02)	(-0.01, 0.03)	(-0.02, 0.02)	(-0.02, 0.02)	(-0.05, 0.02)
p value	0.76	0.24	0.22	0.04	0.33	0.27	0.77	0.85	0.39

[$] Adjusted by sex, age, and participation history for colorectal cancer screening program.

CI: Confidence interval.

P-values were based on the asymptotic standard errors derived from the quantile regression. All analyses were performed using R version 4.3.1.

Q10: Areas with the smallest 10% of uptake difference values; Q90: Areas with the highest 10% of uptake difference values.

Table S2. Coefficients of multivariable quantile regression of all variables on different quantiles of difference in screening interval of colorectal cancer screening program between the period during COVID-19 and the period before COVID-19 among statistical sectors in Flanders, Belgium [$].

Variables	Q10	Q20	Q30	Q40	Q50	Q60	Q70	Q80	Q90
Population density, 1000/km²	0.31	0.16	0.03	-0.08	-0.13	-0.24	-0.39	-0.51	-0.75
95% CI	(0.17, 0.46)	(0.10, 0.22)	(-0.07, 0.12)	(-0.16, 0.00)	(-0.21, -0.04)	(-0.33, -0.15)	(-0.51, -0.27)	(-0.69, -0.32)	(-1.10, -0.39)
p value	<0.001	<0.001	0.61	0.06	<0.01	<0.001	<0.001	<0.001	<0.001
Same address as last year, %	0.12	0.03	0.01	0.03	-0.01	-0.01	-0.01	-0.03	0.06
95% CI	(0.00, 0.24)	(-0.06, 0.13)	(-0.05, 0.08)	(-0.04, 0.10)	(-0.09, 0.07)	(-0.08, 0.05)	(-0.10, 0.08)	(-0.10, 0.04)	(-0.14, 0.26)
p value	0.05	0.49	0.66	0.42	0.78	0.73	0.85	0.43	0.55
Belgian origin nationality, %	0.03	0.01	-0.01	-0.01	-0.01	-0.03	-0.05	-0.05	-0.11
95% CI	(-0.01, 0.08)	(-0.03, 0.04)	(-0.03, 0.02)	(-0.04, 0.02)	(-0.04, 0.01)	(-0.06, 0.01)	(-0.09, -0.01)	(-0.10, -0.01)	(-0.19, -0.03)
p value	0.12	0.72	0.68	0.52	0.31	0.11	0.02	0.02	<0.01
Living alone, %	-0.13	-0.03	-0.05	-0.05	-0.02	0.00	0.02	0.09	0.06
95% CI	(-0.21, -0.04)	(-0.10, 0.04)	(-0.12, 0.02)	(-0.12, 0.01)	(-0.08, 0.04)	(-0.06, 0.07)	(-0.07, 0.11)	(-0.02, 0.19)	(-0.13, 0.24)
p value	<0.01	0.41	0.14	0.11	0.57	0.90	0.65	0.11	0.55
Children in single-parent families, %	0.10	0.04	-0.02	-0.08	-0.03	0.03	0.04	0.08	-0.09
95% CI	(-0.06, 0.26)	(-0.10, 0.17)	(-0.16, 0.12)	(-0.21, 0.05)	(-0.17, 0.10)	(-0.10, 0.17)	(-0.13, 0.22)	(-0.14, 0.29)	(-0.40, 0.22)
p value	0.21	0.58	0.83	0.24	0.60	0.66	0.62	0.49	0.57
Unemployed job seekers, %	-0.32	-0.21	-0.16	-0.06	-0.06	-0.04	0.04	0.10	0.08
95% CI	(-0.52, -0.12)	(-0.34, -0.07)	(-0.31, -0.01)	(-0.20, 0.07)	(-0.20, 0.07)	(-0.16, 0.09)	(-0.14, 0.21)	(-0.12, 0.32)	(-0.32, 0.48)
p value	<0.01	<0.01	0.04	0.34	0.36	0.57	0.70	0.36	0.69
Average net taxable income, 1000€	0.09	0.13	0.19	0.21	0.21	0.24	0.30	0.28	0.42
95% CI	(-0.02, 0.21)	(0.04, 0.23)	(0.10, 0.27)	(0.13, 0.28)	(0.13, 0.29)	(0.15, 0.33)	(0.18, 0.42)	(0.16, 0.41)	(0.21, 0.62)
p value	0.12	<0.01	<0.001	<0.001	<0.001	<0.001	<0.001	<0.001	<0.001
Home ownership, %	-0.05	-0.01	-0.02	-0.01	0.01	0.02	0.04	0.04	0.04
95% CI	(-0.09, -0.01)	(-0.05, 0.02)	(-0.05, 0.01)	(-0.04, 0.02)	(-0.02, 0.04)	(-0.02, 0.05)	(0.01, 0.08)	(-0.01, 0.09)	(-0.05, 0.12)
p value	0.02	0.40	0.23	0.47	0.58	0.33	0.03	0.12	0.38
Social housing per 100 private households	0.00	0.00	0.01	0.01	0.01	0.01	0.00	-0.03	-0.05
95% CI	(-0.03, 0.03)	(-0.02, 0.03)	(-0.02, 0.04)	(-0.02, 0.03)	(-0.01, 0.03)	(-0.02, 0.04)	(-0.02, 0.03)	(-0.07, 0.01)	(-0.12, 0.03)
p value	0.95	0.75	0.40	0.53	0.27	0.41	0.90	0.18	0.24
Cars per 100 households	-0.05	-0.03	-0.03	-0.03	-0.02	-0.01	-0.02	0.01	0.00
95% CI	(-0.08, -0.02)	(-0.05, 0.00)	(-0.05, -0.01)	(-0.05, -0.01)	(-0.04, 0.00)	(-0.04, 0.01)	(-0.05, 0.01)	(-0.02, 0.05)	(-0.06, 0.06)
p value	<0.01	0.02	<0.01	<0.01	0.03	0.28	0.30	0.50	0.95

[$] Adjusted by sex, age, and participation history for colorectal cancer screening program.

CI: Confidence interval.

P-values were based on the asymptotic standard errors derived from the quantile regression. All analyses were performed using R version 4.3.1.

Q10: Areas with the smallest 10% of uptake difference values; Q90: Areas with the highest 10% of uptake difference values.
